# Asthma phenotypes: the intriguing selective intervention with Montelukast

**DOI:** 10.1186/s40733-016-0026-6

**Published:** 2016-08-12

**Authors:** Cottini Marcello, Lombardi Carlo

**Affiliations:** 1 Allergy and Pneumology Outpatient Clinic, Bergamo, Italy; 2 Departmental Unit of Allergology, Immunology & Pulmonary Diseases, Fondazione Poliambulanza, Via Bissolati, 57, 25124 Brescia, Italy

**Keywords:** Asthma, Asthma phenotypes, Montelukast, Asthma therapy, Asthma control

## Abstract

Asthma is a heterogeneous disease, usually characterized by chronic airway inflammation and a variable course associated with various underlying mechanisms that can differ between individuals. Patients with asthma can therefore exhibit different phenotypes, a term used to define the observable characteristics of an organism resulting from the interaction between its genetic makeup and the environment. The heterogeneity of asthma has received a large amount of attention in the last few years in order to better tailor treatment according to the different clinical and biological phenotypes of the disease. Specific asthma phenotypes may require an approach to treatment sometimes different from that recommended by current guidelines, so a personalized approach to asthma pharmacotherapy is recommended. Growing evidence suggests that leukotrienes play an important role in the pathogenesis of bronchial asthma. The mechanisms of action of leukotriene-receptor antagonists theoretically predict a good response in some asthma “phenotypes”.In this article we have performed an analysis of the recent literature (controlled clinical trials and real-life studies) about a possible selective intervention with Montelukast in specific asthma phenotypes.

## Background

**Fig. 1:**
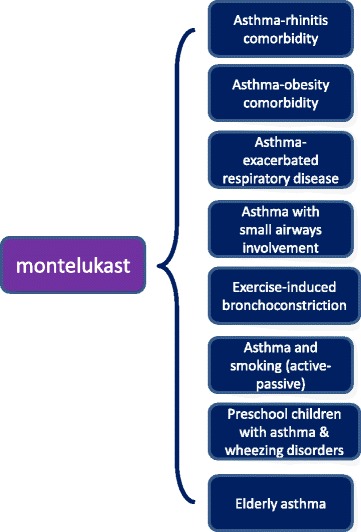
Possibile relationships between Montelukast and asthma phenotypes/endotypes on the basis of controlled clinical trials and real-life studies

Asthma is one of the most common chronic conditions in the world and the most common non-communicable disease among children [1]; according to the Global Burden of Disease Study [2], asthma affects more than 300 million people worldwide. In Europe alone, asthma affects 30 million people [3] and is associated with a significant socioeconomic burden [4]. Although asthma has long been recognized as a heterogeneous disease [5], only in recent years it is seen not as single disease but rather as a series of multiple phenotypes, each defined by an unique interaction between genetic and environmental factors [6]. According to the landmark study of Wenzel [7], phenotype categorizations generally focus on clinical, trigger-related, or inflammatory characteristics, such as eosinophilic, age at onset, treatment-resistant, aspirin-related, obesity-related, or allergic asthma Proposed asthma phenotypes can be further refined into subtypes as defined by a distinct functional or pathophysiological mechanism, which are known as “endotypes” [8]. A precise definition of asthma phenotypes is becoming increasingly important, because recognition of specific sub-phenotypes may further improve our understanding of pathophysiologic mechanisms and treatment response, especially in patients who respond poorly to current therapies [9]. Traditional asthma medications did not work in all patients and there is marked patient-to-patient variability in the therapeutic response; inhaled glucocorticoids (ICS) are used every day, as monotherapy or add-on therapy, by millions of patients with asthma, but about one in three patients may not benefit from this treatment [10]; there is a considerable amount of evidence supporting the concept that some asthma phenotypes seem sensitive to leukotriene receptor antagonists (LTRAs), especially in a real-life setting [11]. Montelukast has proven to be particularly effective in exercise-induced asthma and in asthma associated with allergic rhinitis. Other phenotypes where montelukast is effective include asthma in obese patients, asthma in smokers, aspirin-induced asthma and viral-induced wheezing episodes [12] (Fig. 1). In this article we have performed an analysis of the recent literature (controlled clinical trials, but also pragmatic trials and observational studies, designed to better reflect aspects of routine care than most randomised controlled trials) about the role of Montelukast in asthma therapy, especially in some specific asthma phenotypes.

### Montelukast, a once-daily leukotriene receptor antagonist, in the treatment of chronic asthma

The role of cysteinyl leukotrienes (CysLTs) as key mediators and modulators in the pathogenesis of asthma has been reported in both experimental and clinical settings [13]. CysLTs are produced predominantly by cells of the innate immune system, especially basophils, eosinophils, mast cells, and monocytes/macrophages [14]. The interaction of cysLTs with their type 1 counter-receptors on cells of the innate immune system cause smooth muscle contraction, exacerbate bronchial hyperresponsiveness, impair mucociliary clearance, enhance mucus secretion, increase vascular permeability and also drive Th2- cell-mediated eosinophilic airway inflammation [14].

The bronchospastic effect of CysLTs is greater than that of histamine or methacholine in human airways, both in vivo and in vitro. The CysLTs play an important role in the airway remodeling seen in persistent asthma, that includes increases of airway goblet cells, mucus, blood vessels, smooth muscle, myofibroblasts, and airway fibrosis [15]. Evidence from a mouse model of asthma demonstrated that CysLT1 receptor antagonists inhibit the airway remodeling processes, including eosinophil trafficking to the lungs, eosinophil degranulation, TH2 cytokine release, mucus gland hyperplasia, mucus hypersecretion, smooth muscle cell hyperplasia, collagen deposition, and lung fibrosis [15]. Additional interest in the therapeutic potential of CysLT1 receptor antagonists derives from the facts that neither LT synthesis [16], nor receptor expression [17] is inhibitable by corticosteroids. The recognition of the role of CysLTs in the immunopathogenesis of asthma prompted the development of selective antagonists. Among CysLT1 receptor antagonists, montelukast has shown the best efficacy and safety profile, and it has become the most widely studied antileukotriene compound. Montelukast has been reported to possess secondary anti-inflammatory properties, apparently unrelated to conventional antagonism of cysteinyl leukotriene receptors, suggesting that this agent may have a broader spectrum of anti-inflammatory activities than originally thought [18]. According to results of randomised controlled trials and systematic reviews, Montelukast, when used as monotherapy or added to inhaled corticosteroids, is able to reduce rescue treatment requirements, improve pulmonary function, and reduce symptoms and risk of exacerbations in adults and children with asthma [19, 20]. Guidelines recommend the use of LTRAs as monotherapy for patients with mild persistent asthma, as an alternative or as add-on therapy to ICSs, and as an alternative to either increasing the ICS dose or adding a long-acting 2-agonist [21]. Systematic reviews comparing anti-leukotriene agents and inhaled corticosteroids (ICS) in the management of chronic asthma in adults and children concluded that, as monotherapy, ICS display superior efficacy to anti-leukotrienes (particularly in patients with moderate airway obstruction) [22], endorsed the current guideline position that ICS should be the first-choice preventer therapy in patients initiating maintenance asthma therapy, with leukotriene modifiers as an option [21]. Although RCTs are the cornerstone of evidence-based research, their generalizability to the clinical practice setting is questionable, because recruitment often includes only patients with no (or negligible) co-morbid illnesses or concurrent medications and those with good inhaler technique and high adherence to study therapies, questioning the relevance of their findings for the wide range of patients managed in routine practice [23]; due to their strict inclusion criteria, RCTs exclude approximately 95 % of asthma routine care populations [24]. Real-life studies (e.g., naturalistic or pragmatic clinical trials and observational studies), that include a more heterogeneous asthma population, provide opportunity to explore the interaction between comorbidities, lifestyle factors, patient characteristics and asthma treatment outcomes [11]; real life studies have limitations, primarily stemming from the lack of randomization (the lack of patient selection, one of the most distinctive characteristics of real life studies, makes it impossible to avoid unmeasured confounding factors) and the need to apply the indications only within the local geographic context. Randomized RCTs thus remain the “gold standard” for evaluating treatment outcomes, but observational studies and pragmatic trials can complement classical RCTs by providing data more relevant to the circumstances under which medicine is routinely practiced, providing practical guidance for clinicians [25]. There is growing evidence that efficacy of Montelukast is higher in some asthma phenotypes seem sensitive to Montelukast, especially in a real-life setting [11]. Price et al. in a pragmatic, “real world” study (primary care practices) published in the New England Journal of Medicine, evaluated patients 12 to 80 years of age who had impaired asthma-related quality of life) or inadequate asthma control [26]. This pragmatic trial and health economic evaluation found LTRAs to be equivalent to inhaled corticosteroids at Global Initiative for Asthma (GINA) step 2 and to add-on long-acting β-agonists (LABAs) at GINA step 3. The primary outcome of the trial was a patient-focused quality-of-life outcome, the Mini Asthma Quality of Life Questionnaire at 2 months. No significant differences in Mini Asthma Quality of Life Questionnaire were reported between LTRAs and ICSs (at GINA step 2) or add-on LTRAs and add-on LABAs (at GINA step 3) after 2 months of treatment. Recently, Ann Chen Wu et al. showed that the risk of emergency department visits, hospitalizations, and oral corticosteroids use did not differ between children who initiated leukotriene antagonist and those who initiated inhaled corticosteroid in five health plans and a state Medicaid population [27]. Medication adherence could influence the effectiveness of regimens in practice because adherence to medications in clinical trials is higher than in real-life practice [28]. As with all asthma medications, therapeutic responses to montelukast are highly variable, with some patients responding preferentially to leukotriene modifiers vs other medications [29]; mounting evidence suggests that this heterogeneity in treatment response to montelukast is due, in part, to patient genetics [30]. There is an increasing appreciation of heterogeneity within asthma based primarily on cluster analyses, molecular phenotyping, biomarkers, and differential responses to targeted and non targeted therapies. These studies have led to successful therapeutic trials of molecularly targeted therapies in defined phenotypes (anti-IL-5,anti IL-13,etc) [31]. At the present time, there are no specific biomarkers that can definitively predict responsiveness to anti-LT agents, thus explaining the importance of currently using clinical characteristics to identify responders and the performance of a therapeutic trial in individual patients [32].

### Montelukast: which role in the heterogeneity of asthma?

Patients with specific phenotypes/endotypes, “real-life” comorbidities and lifestyle factors receiving usual care often have different responses to antiasthmatic drugs. The identification of subgroups of asthmatic patients who respond to CysLT [1] receptor antagonists is relevant for asthma management as the response to these drugs is variable [32].

### Asthma and rhinitis

Comorbidity of asthma and rhinitis has been well documented [33], and evidence is accumulating to support more than a coincidental association, suggesting a link between upper and lower airway disease and the hypothesis that asthma and rhinitis represent a systemic disease [34]. Patients with rhinitis have an increased risk of developing asthma [35]. Poorly controlled allergic rhinitis may be associated with worsening asthma control over time [36]. Allergic rhinitis is a very important clinical characteristic of “allergic asthma” endotype [37]. Growing evidence suggests that leukotrienes play an important role in the pathogenesis of allergic rhinitis [38]. A systematic review and meta-analysis of 11 large RCTs showed that leukotriene receptor antagonists were better than placebo and as effective as antihistamines, but less effective than nasal corticosteroids in improving symptoms and quality of life in patients with seasonal allergic rhinitis [39]. Nayak et al. conducted a systematic review of studies that have evaluated montelukast in the treatment of seasonal AR (SAR) and perennial AR (PAR), with and without concomitant asthma. In patients with AR comorbid with asthma, montelukast treatment resulted in significant improvements in both diseases, compared with placebo and allowed a reduction in the use of asthma medication [40]. In the Clinical Outcomes with Montelukast as a Partner Agent to Corticosteroid Therapy (COMPACT) examined whether asthma patients with comorbid allergic rhinitis responded differently to budesonide plus montelukast than patients without comorbid allergic rhinitis in terms of asthma control (lung function). In the subgroup of asthmatic patients with AR, a combined treatment approach that included montelukast and budesonide provided significantly greater efficacy in reducing airflow obstruction compared with doubling the dose of budesonide [41]. Despite the strong association between rhinitis and asthma, patients with rhinitis are often excluded from cRCTs of asthma therapies [42]. Several observational studies evaluate the efficacy and safety of montelukast in patients with both asthma and allergic rhinitis in a real-life setting. In a 12-month, open-label study, Virchow et al. enrolled 1681 patients with mild to moderate asthma insufficiently controlled by ICS or ICS + LABA. Patients received montelukast 10 mg qd as add-on therapy and were evaluated at months 3, 6, 9, and 12. Asthma Control Test (ACT) score in the overall population was the primary endpoint; add-on montelukast demonstrated significant improvement in asthma symptoms over 12 months in all patients in the study, but patients who had allergic rhinitis demonstrated numerically better ACT scores compared with those who did not have allergic rhinitis. The authors concluded that comorbid allergic rhinitis was a strong indicator of better control with add-on montelukast [43] Recently, Ann Chen Wu et al. showed, in five health plans and a state Medicaid population, that children with asthma and allergic rhinitis treated with LTRAs were less likely to experience ED visits (hazard ratio 0.44) compared with the subjects treated with ICS [27]. The results of many “real life” studies suggest that a treatment approach with montelukast, targeting the airway inflammation common to both diseases, may be beneficial for the large proportion of asthma patients who also suffer from allergic rhinitis. In conclusion, treatment guidelines have recognized that asthma and AR are linked conditions of “united airways disease” and recommend that patients with asthma be evaluated for AR and vice versa. These guidelines support a combined approach to treating both conditions [33]. Two International consensus reports concluded that Montelukast may be particularly useful in children when the patient has concomitant rhinitis [44, 45].

### Exercise-induced bronchoconstriction

According to asthma treatment guidelines [21], the presence of activity limitation due to exercise-related symptoms is a key factor for worse asthma control. Exercise-induced bronchoconstriction (EIB) is accompanied by release of mediators such as PGs, CysLTs C4, D4, and E4 and histamine [46]. Several studies with montelukast have shown beneficial effects in adults and children aged as young as 6 years with EIB. The first demonstrations of the efficacy of montelukast in EIB were obtained in the mid-1990s, when the results of studies of the protective effect of montelukast on bronchoconstriction induced by exercise were published [47–49]. It these studies, tolerance was not seen with continued montelukast treatment. The effect of a single dose of montelukast in patients with mild asthma who only have EIB was evaluated in a randomized, crossover, double-blind study study [50]: an initial single dose of montelukast was able to provide significant protection against EIB as soon as 2 h, with persistent benefit up to 24 h. Several randomized control trials specifically evaluated the efficacy of montelukast in EIB in children. In a double blind, placebo-controlled, 3 day doses, crossover study, Montelukast assured protection against exercise-induced bronchoconstriction from the first through the eighth hour from the first day of treatment. However, individual susceptibility to protection was evident since some individuals were not protected at any time [51]. de Benedictis et al. evaluated tolerance to the protective effect of montelukast in exercise-induced bronchoconstriction in children at different time-points over a 4-week treatment period. Montelukast was significantly more protective than placebo against EIB at each time, with no tolerance to the bronchoprotective effect [52]. This aspect is particularly relevant for children, who tend to be active at frequent and irregular intervals throughout the day, and who therefore may benefit from around-the-clock pharmacologic protection. Only few studies have compared regular treatment with Montelukast against that with ICS.Stelmach et al. compared effects of: a) the inhaled ICS budesonide alone, b) budesonide plus the long-acting β2-agonist formoterol, c) budesonide plus montelukast, d) montelukast alone, and e) placebo, in children ages 6–18 with EIB, finding the greatest protection from EIB in either of the two groups given Montelukast [53]. In a crossover study, undertaken in a small group of only 20 patients to compare the ability of both montelukast and budesonide to protect patients from EIB, both budesonide and montelukast significantly reduced the decrease in FEV1 after exercise with respect to the baseline condition of no therapy (*P* = 0.0001). Overall, budesonide offered better protection than did montelukast (*P* = 0.01); however, considerable individual variations in the responses to both budesonide and montelukast were observed [54]. Beta 2-agonists taken immediately before exercise provide significant protection against exercise- induced asthma (EIA) in most patients. However, when they are taken daily, there are some negative aspects regarding severity, control, and recovery from EIB. Daily use of ß2 agonists causes desensitisation of ß2 receptors, leading to enhances mediator release and down regulation of numbers. The mast cell are more affected than muscle, so that duration of protective effect is shorter than the bronchodilator effect [55]. Several studies with LABAs have shown a protective effect on EIB of 10–12 h, but with regular use of LABAs tolerance is common, resulting in a decrease in duration of protection [56, 57]. Several RCTs compared short and long-term protection against EIB of long-acting β2 agonists and Montelukast. Fogel et al. evaluated the effect of montelukast or inhaled salmeterol, added to inhaled fluticasone in reducing FEV1 after a standardized exercise challenge and response to rescue bronchodilation with albuterol in children aged 6 to 14 years with persistent asthma and EIB. The Authors showed that Montelukast, compared with salmeterol, significantly reduced the decrease in FEV1 after exercise and median time to recovery. Response to albuterol rescue after exercise challenge was significantly greater with montelukast [58]. In a double-blind, placebo-controlled study performed at 16 centers in the United States, patients with asthma whose symptoms were uncontrolled on low-dose inhaled fluticasone taking montelukast had significantly greater protection from an exercise-induced decrease in FEV1 than those taking salmeterol (*P* < 0.001). The authors pointed out that concerns in the salmeterol group regarding long-acting β2 -agonists decreased the effects of short-acting β2 -agonists and tachyphylaxis with downregulation of airway smooth muscle receptor numbers [59]. In a comparative systematic review of RCTs in children, the authors conclude that compared with LABAs, LTRAs produce persistent attenuation of EIB and possess an additional effect with rescue short-acting adrenoceptor agonists therapy in asthmatic patients with persistent EIB [60]. It is important to remember that not all patients respond equally. Therefore no matter what treatment is chosen, it is important to have a reevaluation of patient with EIB in 2–4 weeks, as responses vary [61].

### Active smoking

Available information suggest that proportion of patients with asthma who smoke may be similar to that of the general population (20 to 35 %). Increased morbidity and mortality have been reported in asthmatic individuals who smoke [62]. Cigarette smoking among asthmatic patients is associated with worsening symptoms and poorer asthma control [63]. In asthma treatment guidelines, inhaled corticosteroids are considered the standard of care; however, these guidelines are based on evidence from randomised clinical trials that exclude patients who smoke cigarettes and RCT inclusion criteria often resulting in exclusion of not only current smokers, but also any patients with a history of ten pack years or more [64]. Active smoking in asthma is associated with impaired response to corticosteroids [65]. Current asthma guidelines do not provide specific treatment advice for smoking asthmatic patients [64]. The use of LTRAs is a treatment option with potential utility in smoking asthmatics. Evidence has suggested that cigarette smoking induces production of cysteinyl leukotrienes, possibly through COX-1 induction, which could be expected to worsen asthma [66]. Montelukast could be an option in smoking asthmatics, especially if their treatment response to corticosteroids is blunted. Lazarus et al. in a multicenter, placebo-controlled, double-blind, double-dummy, crossover trial, found a blunted response to ICSs, confirming the presence of corticosteroid insensitivity in patients with asthma who smoke; conversely, Montelukast produced a statistically significant increase in a.m. peak flow and a decrease in peak flow variability in smokers, and these changes were significantly greater than its effects seen in nonsmokers [67]. More recently, in a large double-blind asthma RCT involving asthmatic smokers, Price et al. [68] evaluated, 10 mg/day montelukast and 250 mg of medium-dose fluticasone propionate twice daily and placebo. Both 10 mg/day montelukast and 250 mg of fluticasone propionate twice daily significantly increased the mean percentage of days with asthma control compared with placebo. The difference between montelukast and fluticasone was not statistically significant, which contrasts studies in nonsmokers showing superiority of inhaled corticosteroids to montelukast. Smoking history and exposure appeared to play a role in response to therapy: patients with a smoking history of less than the median value of 11 pack years tended to show more benefit of fluticasone, whereas those with a smoking history of greater than 11 pack years tended to show more benefit with montelukast.

### Passive smoking

Many asthmatic patients (particularly children) are exposed to cigarette smoke, known to cause corticosteroid resistance [69]. In asthmatic children, exposure to tobacco smoke (higher cotinine levels) is associated with higher urinary LTE(4) [70]. Rabinovitch et al. followed twenty-seven schoolchildren for 5 months with measurements of urinary leukotriene E4 (LTE(4)), cotinine, fractional exhaled nitric oxide (FENO), and monitored albuterol use. After a baseline run-in, children were randomized to receive daily montelukast or placebo without change in their current controller medications. The authors showed that Montelukast was more effective in children exposed to tobacco smoke, suggesting that the CysLT pathway might play an important role in mediating asthma-related health effects related to secondhand smoke (SHS) exposure [71]. Interestingly, an increased urinary leukotriene E4 level was the only factor that identified the children at high risk for asthma exacerbations.

### Obesity

Obesity has been shown to be risk factor for developing asthma. Obese asthmatics reports worse asthma control despite traditional asthma therapy, worse asthma-specific quality of life, and higher rates of healthcare utilization [72]. Both adult and pediatric studies indicate that obese asthmatics are less responsive to glucocorticoids, the mainstay of asthma controller therapy [73, 74]. Furthermore, obesity is associated with a decreased bronchodilator responsiveness [75]. Obesity is associated with increased expression of 5-lipoxygenase (5-LO) pathway and key members of the LT synthesis pathway are overexpressed in adipose tissue during obesity, resulting in increased LTs levels in this tissue [76]. Increased Leukotrienes production has been observed in obese asthma patients [77]. In a large retrospective analysis of data from four randomized trials comprising 3.037.

Three thousand thirty-seven adults with moderate to severe asthma, Peters-Golden et al. and suggested that obese patients, who are less responsive to ICS than are nonobese subjects, may be relatively more responsive to montelukast. In lean patients, beclomethasone resulted in a higher percentage of asthma control data than did montelukast, at 18.6 % versus 9.5 % (*P* < 0.001). However, the beneficial effect of the inhaled corticosteroid versus the leukotriene modifier became less as BMI increased, with a comparative effect of 18.8 % versus 15.7 % in overweight (*P* = 0.25), and 13.9 versus 13.4 (*P* = 0.90) in obese adults. Compared to ICS, the authors concluded that obese patients with asthma had a better response to montelukast. [78] However, in two other post hoc analyses, comparing ICS or ICS/LABA versus montelukast among the overweight and obese, ICS and ICS/LABA were consistently more effective than montelukast in all BMI catergories [79].

### Aspirin-exacerbated respiratory disease (AERD)

AERD is an “adult-onset” asthma phenotype with high prevalence, associated with chronic hyperplastic rhinosinusitis, nasal polyps, and asthma attacks after ingestion of aspirin and other nonselective COX inhibitors that block COX-1 [80]. The prevalence of AERD in adult asthmatic populations is approximately 10 to 25 % [81]. This asthma phenotype is present most often in patients with severe asthma [82]. Aspirin-exacerbated respiratory disease is explained in part by overexpression of 5-lipoxygenase and leukotriene C4 synthase (LTC4S), resulting in constitutive overproduction of cysteinyl leukotrienes (CysLTs) and driving the surge in CysLT production that occurs with aspirin ingestion [80]. Furthermore, AERD is characterized by the overexpression of CysLT receptors. Subjects with aspirin-intolerant asthma had higher baseline levels of CysLTs in saliva, sputum, blood ex vivo and urine than subjects with aspirin-tolerant asthma [80]. These findings support a global and specific increase in CysLT production in aspirin-intolerant asthma. This asthma phenotype is frequently poorly responsive to inhaled steroids and, as a group, patients with AERD appear to benefit substantially from anti-LT agents. Dahlen et al. investigated whether addition of the leukotriene receptor antagonist montelukast was of therapeutic benefit in a group of 80 aspirin-intolerant patients with asthma of whom 90 % already were treated with moderate to high doses of glucocorticosteroids [83]. The group receiving montelukast showed a remarkable improvement of their asthma, whereas the group given placebo showed no change. The improved pulmonary function in the group receiving montelukast was associated with fewer asthma symptoms and fewer asthma exacerbations. Mastalerz et al. compared the clinical response to montelukast in aspirin-intolerant asthmatics (AIAs) and aspirin-tolerant asthmatics (ATAs). Following a 3-week montelukast 10 mg day-1 treatment compared with placebo, both groups showed a similar significant improvement in asthma control, morning and evening peak expiratory and quality of life [84]. Recently, a survey analyzed perceptions and quality of life in patients living with AERD and queried patient observations of treatment effectiveness [85]. Patients do not appear to be satisfied with current treatment options as evidenced by persistent symptoms, adverse effect on quality of life, and pursuit of various alternative treatment options. Of all the treatments offered, aspirin desensitization was reported to be the most beneficial, followed by a leukotriene receptor antagonist and a combination of medications. In this survey, half of the respondents on a leukotriene receptor antagonist found it to be helpful. Micheletto et al. [86] studied 36 nonsmoker subjects with AIA and performed a nasal provocation test with lysine-aspirin (L-ASA) in baseline and after a 4-week Montelukast 10 mg or placebo treatment and showed that Montelukast, but not placebo, improves nasal function and nasal response to Aspirin substantially in ASA-sensitive asthmatics. In conclusion, the results of these studies and the role of constitutive overproduction of cysteinyl leukotrienes in the pathogenesis of disease, support the view that treatment with montelukast, generally as add-on therapy, can improve asthma control and nasal symptoms in asthmatic patients with AERD.

### Asthma in elderly patients

Recent epidemiologic studies have indicated that asthma is highly frequent in the elderly population with a prevalence ranging from 4.5 to 12.7 % [87]. Compared to children or younger adults, older adults and/or elderly subjects have greater morbidity and healthcare costs from asthma. In a study by Tsai et al. [88], elderly subjects (ages 65 years and older) had fourfold greater overall mortality than subjects ages 18 to 64.9 years. Therapeutic approach to asthma in elderly patients does not differ from what is recommended for young patients, but there have been few reports regarding the efficacy of therapy in older patients, and most recommendations are an arbitrary extrapolation of what has been tested in younger subjects [89]. Montelukast could be of interest in the treatment of asthma in the elderly, as it could contribute to obtain symptom control by enhancing patients’adherence, frequently reduced in the elderly [90]. Elderly patients have usually multiple comorbid conditions, and they are also prone to have poor inhaler technique/adherence combined with lack of caregivers and cognitive impairment [91]. Inhaler misuse is common in the real-world setting with both pressurized metered dose inhalers (pMDIs) and dry powder inhalers (DPIs), and it is associated with poor asthma control [92]. In elderly patients, the simpler route of administration of Montelukast, compared with the inhaled agents, could represent a more effective strategy in improving the outcomes of asthma therapy, given that unintentional nonadherence with inhalation therapy may lead to significant impairment of asthma symptom control [93]. Recently, Ye et al. compared, in a randomized, open-label, parallel-designed trial, the efficacy of the addition of montelukast to low-dose inhaled budesonide (MON-400 BUD) versus increasing the dose of inhaled steroid (800 BUD) on asthma control in older asthmatics. The efficacy of 12-week treatment with MON-400 BUD in older asthmatics was comparable to that of 800 BUD on asthma control but associated with reduced frequency of asthma exacerbations requiring oral steroids and sore throat events [94]. Bozek et a. [95] evaluated 512 elderly patients (>60 years old) with severe asthma over 24 months of therapy: the first 12 months using inhaled corticosteroids (ICS) and long-acting beta-agonists (LABA) and the second 12 months with oral montelukast added in two-thirds of the patients, with the remaining third representing the control group. During the first year of treatment using ICS and LABA, an increase in the median percentage of days without asthma was observed, as well as a decrease in the percentage of days with short beta-receptor agonist use. These differences were significantly greater when montelukast was added to the therapy (78.4 and 39.5 %, respectively). This improvement was not observed in the control group. Leukotriene modifiers have been demonstrated to be safe in elderly asthmatic, even though cases of acute hepatitis and occurrence of Churg-Strauss syndrome have been described; whether this is associated with age is to be confirmed [89]. Taken together, these observations are reassuring regarding safe use and efficacy of montelukast in the management of asthma at advanced ages, as an add-on treatment to inhaled combination therapy to control the disease or as an alternative to inhaled corticosteroids or long-acting beta-2 agonists when these are contraindicated in the elderly population.

### “Small airways” phenotypes

Recent studies suggest that persistent uncontrolled inflammation in the peripheral small airways can also contribute to clinical expression and worse control of asthma, with increased asthma symptoms, more severe bronchial hyper-responsiveness and an increased number of exacerbations [96]. These findings support the view that distal lung is a very important target in any therapeutic strategy for effective treatment. Several studies have assessed the ability of both inhaled small particle aerosols and oral treatments to target the distal airways and improve physiological indices and levels of asthma control. Montelukast is a systematically administered leukotriene receptor antagonist that reaches the small and large airways [97]. Leukotriene receptors are differently expressed in fibroblasts from peripheral compared to central airways [98], which may explain a suggested cysteinyl-leukotriene driven remodeling mainly in the peripheral airways and possibly resulting in a predominant effect of montelukast on the small airways. Mechiche et al. reported that the CysLTs were about 30-fold more potent in small bronchi than in larger bronchi and that Montelukast exerts a potent antagonist activity against the particularly potent constricting effects of CysLTs in isolated human small bronchi [99]. Several studies showed that biomarkers of peripheral airways are improved by montelukast: peripheral airways resistance [100], air trapping [101], and alveolar nitric oxide [102]. There is suggestive evidence that a improvement in distal dysfunction/inflammation after treatment with montelukast is associated with better asthma control and asthma-related Quality of Life [103].

### Preschool children with asthma and wheezing disorders

Wheezing and shortness of breath in preschool children are among the most common presenting symptoms in paediatric practice. Approximately 50 % of children experience a wheezing illness during the first 6 years of life, and one-third of young children from the United States and Europe experienced multiple days troubled by cough, wheeze, or breathlessness over the preceding 6 winter months [104]. Although about two-thirds of these children lose their symptoms after the age of 6 years, the disease places a considerable burden on the child, the child’s family, and society because of the high prevalence and lack of good treatment control [105]. While early transient wheeze is a benign condition, with no sequelae for respiratory health by age 18, intermediate-onset and persistent wheeze phenotypes are associated with reduced growth in pre-bronchodilator FEV1 over adolescence and sometimes with irreversible airflow limitation by 18 years [106]. Viral infections account for up to 85 % of childhood asthma exacerbations, daily symptoms, and exacerbations in children with asthma [107]. Since 2008, phenotypical classifications proposed by international paediatric groups, based on the temporality of symptoms (episodic or persistent) and asthma triggers (viral only, allergens, exercise, or multitriggers), have been recommended to better support therapeutic decisions [108]. The typical wheezing pattern in infants and preschool aged children consists of short but recurrent exacerbations of cough and wheeze triggered by viral infections and separated by long symptom-free intervals [109]. A ERS Task Force report [110] recommended distinguishing between two phenotypes based on temporal patterns of wheeze:episodic viral wheeze (EVW, wheezing during discrete time periods, often in association with clinical evidence of a viral cold, with absence of wheeze between episodes) and multiple-trigger wheeze (MTW, wheezing that shows discrete exacerbations, as with episodic viral wheeze, but also symptoms between episodes). Both daily ICS and LTRA therapies have shown efficacy in the management of intermittent wheezing in preschool children, and intermittent high-dose ICS therapy is comparable in efficacy to daily low-dose ICS therapy in high risk children [109]. Preschool children with a history of episodic wheezing, who are at high risk for asthma, but without evidence of day-to-day impairment, have improved clinical courses in terms of exacerbations requiring OCS when they receive ICS therapy, either as a daily low-dose ICS regimen or as an intermittent high-dose ICS [104, 111]. Despite the established efficacy of daily low-dose ICS in increasing episode-free days, parental adherence to such approaches in clinical care is suboptimal, likely due to the episodic nature of the disease in preschool children and concerns surrounding the safety of ICS therapy [104]. The excellent safety profile of montelukast, and the possibility of oral administration, which entails better compliance from young children, represent the main strengths of its use in preschool children. Therefore, montelukast represents an alternative to ICS in poorly compliant preschool children, or in subjects who show adverse effects related to long-term steroid therapy [112]. Furthermore, evidence suggests that leukotrienes play a key role in viral-induced respiratory illness [113]. Leukotriene*s* can be detected up to 28 days after the onset of viral-induced respiratory illness, suggesting the need for long-term treatment. Clinical trial data in preschool-aged children with persistent asthma demonstrate that daily use of montelukast for 12 weeks significantly reduces asthma symptom frequency, rescue albuterol use, oral corticosteroid use, and peripheral blood eosinophil counts [114]. Szefler et al. compared budesonide inhalation suspension (BIS) and montelukast over a 1-year period in 202 children aged 2 to 4 years with mild persistent asthma. BIS and montelukast provided acceptable asthma control, with no significant difference between treatments in the primary end point; however, several secondary outcomes showed statistically significant differences in favor of BIS over montelukast [115]. Among 26 preschool-aged children with mild asthma, montelukast therapy over a 4-week period was associated with a 2.5-fold reduction in bronchial hyperresponsiveness (BHR) to methacholine relative to placebo [116]. Initiation of open label montelukast in preschool-aged children with persistent asthma and fraction of exhaled nitric oxide levels of 10 ppb or greater was associated with a significant decrease in fraction of exhaled nitric oxide levels, along with improvements in BHR to adenosine, lung function (by means of forced oscillation), and symptom scores over an 8-week period [117] Finally, one study of 194 children (22 % aged 2 to 5 years) showed that montelukast added to the usual treatment with ICS reduced the risk of worsening asthma symptoms (53 % less) and unscheduled physician visits (78 % less) during the annual September asthma epidemic [114]. Boys aged 2 to 5 years showed greater benefit from montelukast than did older boys [118]. The GINA and NAEPP/EPR3 guidelines identify ICSs as the preferred controller at step 2, with montelukast identified as an alternative in children 0 to 5 years of age [21, 119]. In a recent report, an international consensus group reviews the new evidence and proposes some modifications to the recommendations made in 2008 [120]; there was consensus that ICS are the first-choice maintenance therapy for MTW,while, in EVW with severe or frequent attacks, either ICS or montelukast may be prescribed. Recently [121], a Cochrane Database Systematic Review, mainly based on patients in intermittent therapy, concluded that, in pre-school children with EVW, there is no evidence of benefit associated with maintenance or intermittent LTRA treatment, compared to placebo, for reducing the number of children with one or more viral-induced episodes requiring rescue oral corticosteroids, and little evidence of significant clinical benefit for other secondary outcomes. However, the authors acknowledge that children with an apparent EVW phenotype are not a homogeneous group and that subgroups may respond to LTRA treatment depending on the patho-physiological mechanisms involved and the genetic background. Recently, Nwokoro et al. [122] showed no clear benefit of intermittent montelukast in young children with wheeze. However, the 5/5 ALOX5 promoter genotype might identify a montelukast-responsive subgroup. In conclusion, the decision to start any controller therapy in preschool children is most strongly determined by the pattern, frequency and severity of symptoms. [104] Any preschool child with troublesome recurrent wheeze could be started on either ICS (first choice) or montelukast [108, 109].

### Safety

Montelukast is generally considered a safe drug with the occurrence of a few adverse drug reactions (ADRs). The overall incidence of ADRs due to montelukast, based on clinical data, suggests that it is comparable to placebo and its use as add-on therapy does not seem to increase ADRs in comparison to therapy based on ICS or beta-2 stimulants. Recently, a Systematic Review and Meta-Analysis compared the efficacy and safety of LTRAs with placebo in adults and adolescents [19]. The proportions of patients with adverse events were generally similar in the intervention and comparator groups. Across all trials, no serious adverse events were reported. Five trials explicitly reported no adverse events. The Authors concluded that the incidence of adverse events and withdrawals due to adverse events and worsening asthma was similar for LTRAs and placebo, which reflects a favorable safety and tolerability profile for LTRAs. A review of clinical trials summarized the safety and tolerability information for montelukast evaluating data from 2751 paediatric patients (preschool and school children). Montelukast was well-tolerated, and the most frequent clinical ADRs noticed in all treatments (placebo, montelukast and active control/usual care) in virtually all studies were upper respiratory infection, worsening asthma, pharyngitis, and fever [122]. There is conflicting evidence regarding the association between montelukast and neuropsychiatric events (NE). Recently, Ali et al. examinated this association among children with asthma and 1920 subjects less than 18 years of age with a primary diagnosis of asthma between 1 January 1998 and 31 December 2009 were identified. A clear dose-response relationship was not observed, with an adjusted OR of 1.01 (95 % CI [0.88, 1.14] for experiencing NE [123, 124].

## Conclusions

In summary, evidence from real-life studies and randomised controlled trials, show that montelukast (the most widely used of the LTRAs) is effective on many biological and pathophysiological mechanisms involved in asthma. Montelukast, when used as monotherapy or added to inhaled corticosteroids, is able to reduce rescue treatment requirements, improve pulmonary function, and reduce symptoms and risk of exacerbations in adults and children with asthma. Poor adherence to ICS is common and contributes to worse control of asthma, severe exacerbations, including hospitalizations and emergency department visits. The simpler route of administration of Montelukast, compared with the inhaled agents, could represent a more effective strategy in improving adherence to asthma therapy. There is a considerable amount of evidence supporting the concept that some asthma phenotypes seem sensitive to montelukast, especially in a real-life setting. Montelukast has proven to be particularly effective in exercise-induced asthma and in asthma associated with allergic rhinitis. Other phenotypes where montelukast is effective include asthma in obese patients, asthma in smokers, aspirin-exacerbated respiratory disease and viral-induced wheezing episodes. At the present time, there is no phenotypic feature or routinely available laboratory measure that can definitively predict responsiveness to anti-LT agents, necessitating the performance of a therapeutic trial in individual patients. Montelukast is generally considered a safe drug with the occurrence of a few adverse drug reactions.
